# Effectiveness and safety of acupuncture for anxiety disorder of coronavirus disease 2019: A protocol of systematic review and meta-analysis

**DOI:** 10.1097/MD.0000000000022177

**Published:** 2020-09-18

**Authors:** Yu Zhang, Ting Ren, Hongyu Li, Luwen Zhu, Qiang Tang

**Affiliations:** a Department of Rehabilitation, Heilongjiang University of Chinese Medicine, Harbin, China; b Department of Rehabilitation, The Second Affiliated Hospital of Heilongjiang University of Chinese Medicine, Harbin, China.

**Keywords:** acupuncture, anxiety, COVID-19, protocol, systematic review

## Abstract

**Background:**

Anxiety disorder places a heavy burden in the clinical treatment of patients of COVID-19. Acupuncture is a recommended treatment of COVID-19 in China, and clinical researches showed the effectiveness of acupuncture. We will conduct this systematic review and meta-analysis to assess the effectiveness and safety of acupuncture for COVID-19.

**Methods:**

Electronic databases of Medline, EMBASE, Cochrane Library, China National Knowledge Infrastructure (CNKI), Chinese Biomedical literature Database (CBM), Chinese Scientific and Journal Database (VIP), and Wan Fang database (Wanfang) will be searched for randomized controlled trials of acupuncture for anxiety disorder of COVID-19 from inception of the database to August 10, 2020. Two reviewers will screen studies, collect information independently. We will utilize RevMan 5.3 for meta-analysis.

**Results:**

We will publish the study result to a peer-reviewed journal.

**Conclusion:**

This study will contribute to provide high-quality evidence of acupuncture for anxiety disorder of COVID-19

## 1 Introduction

In December 2019, infectious diseases, characterized by fatigue, fever, cough, breathing difficulties, chest pain, headache, muscle pain, gastrointestinal discomfort, etc, were spreading widely in Wuhan, China.^[1–3]^ In January 2020, the World Health Organization (WHO) announced that the epidemic lead to SARS-CoV-2, which was found in one patient's pharynx swab sample was called the Coronavirus Disease 2019 (COVID-19).^[4]^ There have been more than 13 million people worldwide confirmed with 2019 coronavirus (COVID-19) and more than 580,000 deaths as of July 16, 2020. Because of its high infectious, huge harmfulness, and wide extensiveness, COVID-19 has been proclaimed as a public health emergency of international concerned by the WHO.^[5,6]^ Investigation reveals that susceptibility, harmful consequence, and mortality of COVID-19 may be related to age, gender, and personal usual health condition: people with items of older age, male, and serious basic health conditions are more susceptible to it.^[7,8]^

China published guidelines and treatment instruction of COVID-19, and recommended using acupuncture as adjuvant treatment.^[9]^ Acupuncture was used for anxiety disorder of COVID-19 in China; however, a high-quality evidence for the acupuncture treatment has been still lacking for it. Thus, we conduct this study to assess the effectiveness and safety of acupuncture for breathlessness of COVID-19.

## 2 Methods

### 2.1 Study registration

This systematic review and meta-analysis was registered on PROSPERO (CRD42020202258), and it will be performed according to Preferred Reporting Items for Systematic Review and Meta-Analysis Protocols (PRISMA-P) 2015 statement.^[10]^

### 2.2 Inclusion criteria

#### 2.2.1 Types of participants

Patients of coronavirus disease 2019 with positive nucleic acid test will be included, regardless of gender and race.

#### 2.2.2 Types of intervention

The intervention of the experimental group contains acupuncture, which included warm acupuncture, electroacupuncture, scalp-acupuncture, hydroacupuncture, and manual acupuncture. The control group could use any kinds of treatments, which may be western medicine, Chinese herbal medicine, placebo, or regular treatment, except acupuncture.

#### 2.2.3 Types of outcomes

##### 2.2.3.1 The primary outcome

Hamilton Anxiety Scale (HAM-A)^[11]^ was created in 1959 and has been used for measuring the severity of perceived anxiety symptoms till today, is also considered one of the most widely used rating scales.

##### 2.2.3.2 Second outcomes

The Liebowitz Social Anxiety Scale (LSAS),^[12]^ which is the most frequently used for assessing social anxiety disorder (SAD) in clinical research and practice; (modified) Barthel index (MBI),^[13]^ is usually used for measuring individual's performance on activities of daily living (ADL) functions.

Side effect or adverse event of included studies will be utilized for safety analysis.

#### 2.2.4 Types of studies

We will include randomized controlled trials (RCTs).

### 2.3 Search methods

We will search electronic databases of Medline, EMBASE, Cochrane Library, China National Knowledge Infrastructure (CNKI), Chinese Biomedical literature Database (CBM), Chinese Scientific and Journal Database (VIP), and Wan Fang database (Wanfang) for RCTs of acupuncture for anxiety disorder of COVID-19 from inception of the database to August 10, 2020. The search strategy of Medline is given in Table 1.

**Table 1:**
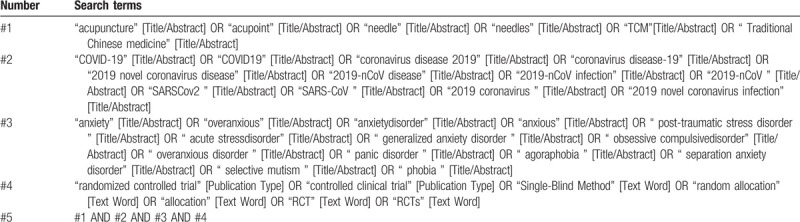
Search strategy for Medline.

### 2.4 Selections of studies

**Figure 1:**
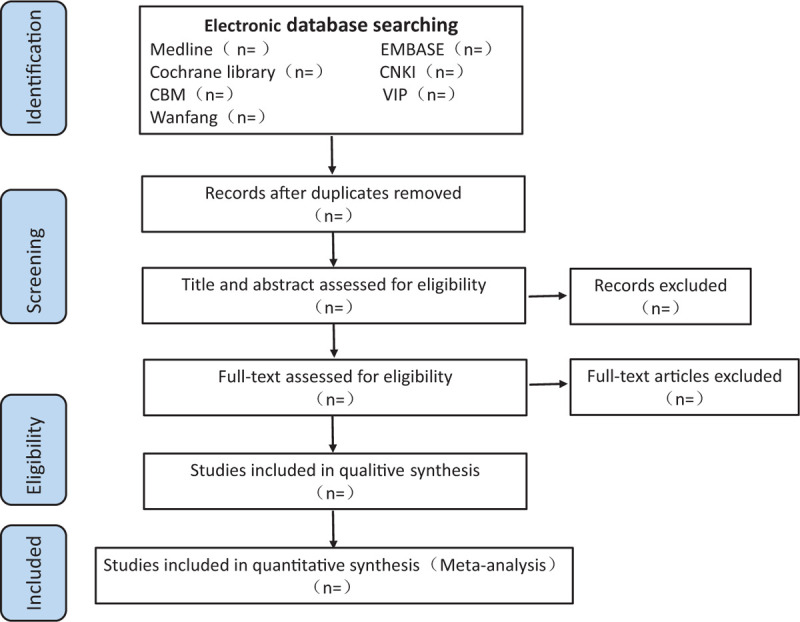
The selecting process.

Two reviewers will select studies. First, they will remove the duplicates, and then they will eliminate studies, which are irrelevant though reading titles and abstracts. Then, they will read the full text of the rest literature for further screening. (Fig. 1 shows the selection process); the selection process will be performed by 2 reviewers according to the inclusion and exclusion criteria independently.

### 2.5 Data collection and management

Two reviewers will select studies and extract data according to screening process. A form designed in advance would be made used of extracting data, which including title, authors, publication year, publication journal; patients’ information of gender, age, number of each group, diagnostic criteria, intervention of each group, and major and second outcomes. The operation of selecting literature and extracting information will be done by 2 reviewers independently. They will refer to another reviewer, if disagreements come.

### 2.6 Assessment of risk bias

Cochrane Risk of Bias Tool according to the Cochrane Handbook 5.1.0 for Systematic Reviews of Interventions includes 7 items: the risk of bias of sequence generation, allocation concealment, blinding of participants personnel and outcome assessment, incomplete outcome data, selective outcome reporting and other bias, will be used for assessing the risk of bias by 2 reviewers independently, the grades of evaluation will be low, unclear risk, and high risk of bias. Third reviewer will be referred to, if there would be any disagreement.

### 2.7 Data syntheses

#### 2.7.1 Measures of treatment effect

We will use standard mean differences (SMDs) or weighted mean differences (WMDs) with 95% confidence intervals (95% CIs) to analyze continuous outcomes for assessing acupuncture in the treatment of anxiety disorder of coronavirus disease 2019.

#### 2.7.2 Assessment of heterogeneity and data synthesis

RevMan software 5.3.5 will be utilized for statistical analyses. Statistical heterogeneity will be evaluated with *I*^2^, when *I*^2^ < 25% indicates negligible heterogeneity, 25% ≤ *I*^2^ < 50% indicates moderate heterogeneity; however, *I*^2^ ≥50% means high heterogeneity. Fixed-effect model will be used when heterogeneity below moderate (*I*^2^ < 50%; *P* > .1); on the contrary, random-effects model will be chose when heterogeneity is high. We will analyze continuous variables with SMDs or a WMDs with 95% CIs.

#### 2.7.3 Assessment of reporting bias

We will utilize funnel plot for assessing the reporting bias if the included literatures are more than 10. If the funnel plot is symmetrical, then it means there is no publishing bias. However, not symmetrical indicates that there exits publishing bias. We may turn to *P-*value if included studies are less than 10.

#### 2.7.4 Subgroup analysis

If significant heterogeneity exits, we will perform subgroup analysis according to different types of acupuncture and different course of treatment.

#### 2.7.5 Sensitivity analysis

We will conduct the sensitivity analysis for assessing the robustness and reliability of the results by excluding low-quality studies and focusing on missing data.

#### 2.7.6 Grading the quality of evidence

We will make use of Grading of Recommendations Assessment, Development, and Evaluation Reliability Study (GRADE) for assessing the quality of evidence.

#### 2.7.7 Dealing with missing data

We will try to contact the corresponding author through e-mail for obtaining the missing information we needed. If it does not work, we will make use of the available data for synthesis, and the potential impact of missing information will be reviewed.

#### 2.7.8 Ethics and dissemination

No ethical approval is needed in this study, for nothing of the data will be obtained from an individual patient. We plan to publish this systematic review and meta-analysis in a peer-reviewed journal.

## 3 Discussion

The COVID-19 pandemic presented huge physical and mental stress for patients who received a definite diagnosis, for most of them were isolated for treatment after diagnosis and could not be accompanied by their families and friends. Anxiety,^[14,15]^ which is one of a growing number of psychological problems, has been increasingly affecting patients’ daily life and normal treatment. Acupuncture is a traditional Chinese medicine external treatment, which has been used for thousands of years. Studies^[16–18]^ show that acupuncture has a good effect for anxiety. This systematic review and meta-analysis was conducted to assess the efficiency and safety of acupuncture for anxiety disorder of COVID-19 and provide a traditional medicine treatment for clinical decision.

## Author contributions

**Conceptualization:** Qiang Tang, Yu Zhang

**Data curation:** Yu Zhang, Ting Ren

**Formal analysis:** Yu Zhang, Luwen Zhu

**Funding:** Qiang Tang

**Investigation:** Luwen Zhu, hongyu li

**Methodology:** Yu Zhang, Qiang Tang

**Project administration:** Qiang Tang

**Resources:** Yu Zhang

**Software:** Yu Zhang

**Writing – original draft:** Yu Zhang

**Writing – review & editing:** Qiang Tang, Luwen Zhu

## Abbreviations

Abbreviations: ADL = activities of daily living, CBM = Chinese Biomedical literature Database, CIs = credible intervals, CNKI = China National Knowledge Infrastructure, HAM-A = Hamilton Anxiety Scale, LSAS = The Liebowitz Social Anxiety Scale, MBI = (Modified) Barthel index, MDs = mean differences, PRISMA-P = Preferred Reporting Items for Systematic Review and Meta-Analysis Protocols, RCTs = Randomized Clinical Trails, SAD = social anxiety disorder, SMDs = standard mean differences, VIP = Chinese Scientific and Journal Database, Wanfang = Wan Fang database, WOS = Web of Science.
